# Classical Activation of Macrophages Leads to Lipid Droplet Formation Without *de novo* Fatty Acid Synthesis

**DOI:** 10.3389/fimmu.2020.00131

**Published:** 2020-02-18

**Authors:** Mauricio Rosas-Ballina, Xue Li Guan, Alexander Schmidt, Dirk Bumann

**Affiliations:** ^1^Focal Area Infection Biology, Biozentrum, University of Basel, Basel, Switzerland; ^2^Lee Kong Chian School of Medicine, Nanyang Technological University, Singapore, Singapore; ^3^Proteomics Core Facility, Biozentrum, University of Basel, Basel, Switzerland

**Keywords:** lipid metabolism, macrophage activation, lipid droplet (LD), beta-oxidation, interferon, inflammation

## Abstract

Altered lipid metabolism in macrophages is associated with various important inflammatory conditions. Although lipid metabolism is an important target for therapeutic intervention, the metabolic requirement involved in lipid accumulation during pro-inflammatory activation of macrophages remains incompletely characterized. We show here that macrophage activation with IFNγ results in increased aerobic glycolysis, iNOS-dependent inhibition of respiration, and accumulation of triacylglycerol. Surprisingly, metabolite tracing with ^13^C-labeled glucose revealed that the glucose contributed to the glycerol groups in triacylglycerol (TAG), rather than to *de novo* synthesis of fatty acids. This is in stark contrast to the otherwise similar metabolism of cancer cells, and previous results obtained in activated macrophages and dendritic cells. Our results establish a novel metabolic pathway whereby glucose provides glycerol to the headgroup of TAG during classical macrophage activation.

## Introduction

Activation of macrophages with pro-inflammatory stimuli, also known as classical M1 activation, induces a profound shift in energetic metabolism characterized by aerobic glycolysis and decreased mitochondrial substrate oxidation (1, 2). Lipid accumulation is another salient metabolic feature of phagocyte activation during infection and sterile inflammation (3, 4). During these conditions, lipids accumulate in single membrane organelles known as lipid droplets or lipid bodies (5–7). Lipid droplets originate from endoplasmic reticulum and contain a core of neutral lipid, namely triacylglycerol (TAG) and cholesterol ester (8–10). Lipids contained within lipid droplets can be used as substrate for ATP synthesis through β-oxidation and as precursors for membrane lipids, eicosanoids, and nuclear receptor ligands (11–14).

While lipid accumulation in phagocytes is a hallmark of infection and sterile inflammation, its underlying biosynthesis pathways are still unclear. Tracing studies of radiolabeled substrate incorporation into total cellular lipids suggest that *de novo* fatty acid synthesis from glucose contributes to lipid accumulation in macrophages in murine models of sterile inflammation (15, 16), and in classically-activated macrophages and dendritic cells *in vitro* (11, 16, 17). However, this approach does not provide information regarding the site of carbon incorporation, i.e., lipid headgroup vs. fatty acid. On the other hand, lipids contained in lipoproteins are taken up by macrophages leading to the formation of cytoplasmic lipid inclusions characteristic of “foam cells” in the atherosclerotic plaque (18, 19). Thus, the question remains as to whether lipids accumulating during classical macrophage activation originate from *de novo* fatty acid synthesis or from an exogenous source of lipid.

We show here that activation of macrophages with interferon gamma (IFNγ), a major mediator of sterile and bacterial-induced inflammation, increases glucose uptake and lactate release. Further, IFNγ increases total TAG levels, and induces lipid droplet accumulation that depends on exogenous lipids. Metabolite tracing with ^13^C-labeled substrates revealed that *de novo* synthesis of fatty acid from glucose plays a minor role, if at all, in TAG accumulation. Rather, glucose provides to the glycerol headgroup of TAG, while the acyl chains of TAG originate from exogenous fatty acid (FA). Finally, we show that nitric oxide produced by inducible nitric oxide synthase (iNOS) inhibits mitochondrial respiration and therefore oxidation of FA, which instead accumulates in lipid droplets.

## Results

### Maf-DKO Cells Polarize to M1 and M2 Phenotypes

In order to study the metabolic basis of lipid droplet accumulation, we used IFNγ to activate MafB/c-Maf double deficient (Maf-DKO) primary mouse macrophages. These cells are a bona fide alternative to other macrophage sources such as RAW cells as they are not transformed cells with distorted metabolism typical of cancer cells; maintain a differentiated macrophage phenotype when expanded in culture; and functionally integrate into tissues without causing tumors when transplanted into mice (20, 21). Activation with IFNγ led to expression of inducible nitric oxide synthase (iNOS) and production of TNF whereas IL-4 led to arginase-1 expression and failed to induce TNF production (Figures S1A,B). IFNγ also increased the expression of the class II major histocompatibility (MHC II) molecule I-A/I-E and CD86 (Figures S1C,D) consistent with classical M1 macrophage activation (22–24). Thus, Maf-DKO cells polarize to M1 and M2 phenotypes when activated with IFNγ and IL-4, respectively.

### IFNγ Induces Lipid Droplet and Triacylglycerol Accumulation

**Figure 1:**
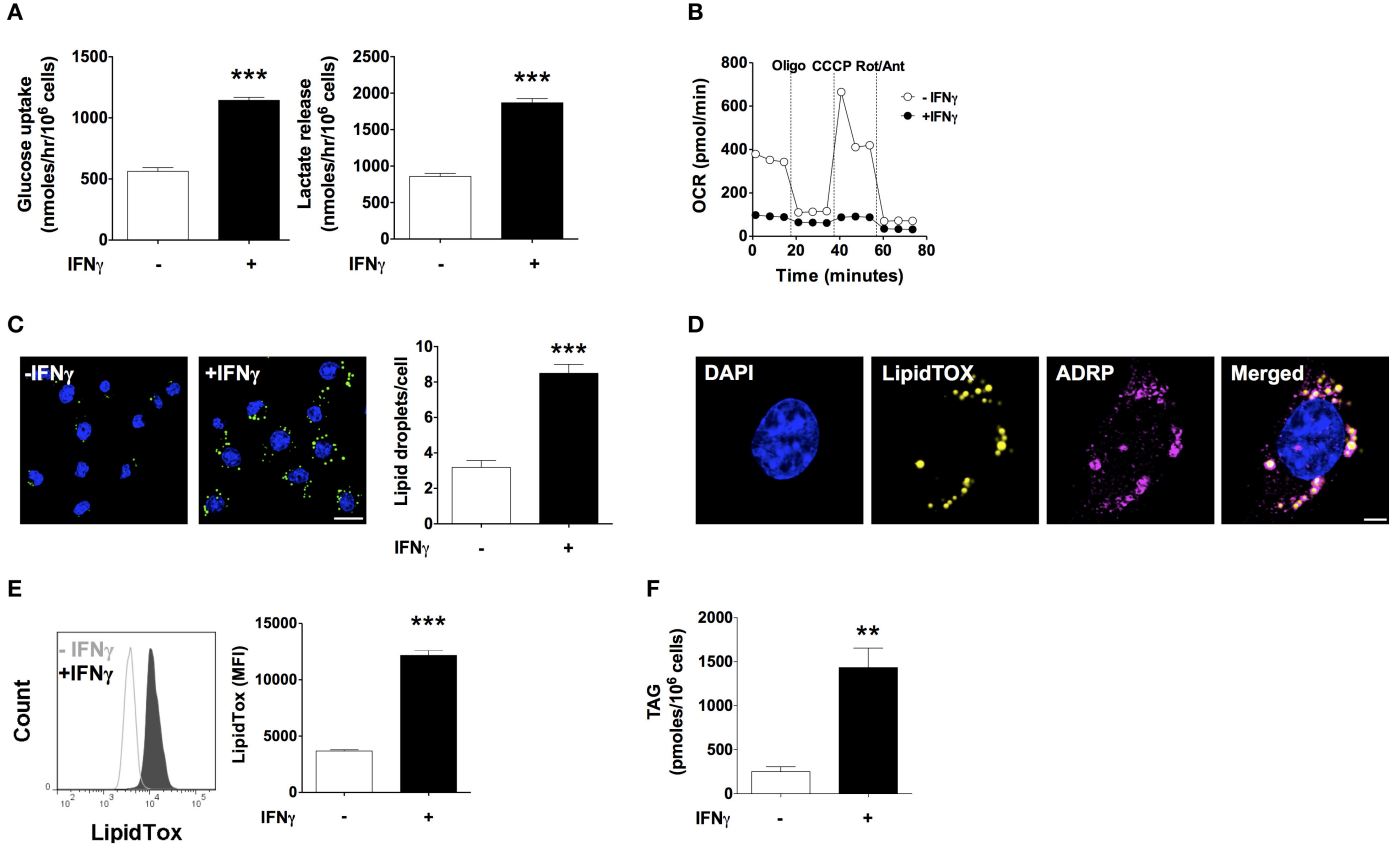
IFNγ up-regulates glucose uptake and lactate release, inhibits respiration, and induces triacylglycerol and lipid droplet accumulation. Maf-DKO macrophages were incubated for 24 h in the presence or absence of IFNγ (10 ng/mL). **(A)** Glucose uptake and lactate release rates were obtained after 24 h of incubation with or without IFNγ by measuring metabolite concentration in supernatants sampled hourly over a period of 3 h. Data shown as mean ± SEM of three replicates. Data is representative of three experiments. **(B)** OCR under basal conditions and after sequential addition of oligomycin (Oligo), carbonyl cyanide m-chlorophenyl hydrazine (CCCP), and rotenone plus antimycin (Rot/Ant). Data shown as mean ± SEM of six replicates. Data is representative of three experiments. **(C)** Confocal fluorescence image of cells stained with LipidTOX and DAPI, and quantification of LipidTOX-positive organelles. Data shown as mean ± SEM of three replicates from a single experiment. Scale bar 12.5 μm. **(D)** Confocal fluorescence image of cells activated with IFNγ stained with DAPI, LipidTOX, and anti-ADRP (perilipin 2) antibody. Scale bar 2.5 μm. **(E)** Histogram and quantification of the mean fluorescence intensity (MFI) of cells stained with LipidTOX. Data shown as mean ± SEM of three replicates. Data is representative of three experiments. **(F)** Total cell triacylgylcerol (TAG) content. Data shown as mean ± SEM of four replicates from a single experiment. **p < 0.01; ***p < 0.001.

IFNγ induced a 2-fold increase in glucose uptake rate and a 2-fold increase in lactate release rate (Figure 1A). Moreover, oxygen consumption rate (OCR) decreased by 75% with IFNγ. Inhibition of ATP synthase with oligomycin reduced oxygen consumption in non-activated macrophages, indicating coupling of oxygen consumption with ATP production. Instead, oligomycin barely reduced the already decreased OCR in macrophages activated IFNγ indicating that mitochondria were producing few or no ATP. In non-activated macrophages, uncoupling of oxidative phosphorylation from ATP synthesis with the ionophore carbonyl cyanide 3-chlorophenylhydrazone (CCCP) increased OCR, as expected in cells with intact mitochondrial function in order to maintain the mitochondrial membrane potential. The difference between the basal OCR and CCCP-induced increase in OCR (spare respiratory capacity) was completely abolished in macrophages activated with IFNγ, suggesting mitochondrial dysfunction (Figure 1B). Staining with LipidTOX, a fluorescent dye specific to neutral lipids, showed round cytoplasmic organelles whose numbers almost tripled upon activation with IFNγ (Figure 1C). These LipidTOX-positive organelles were surrounded by the adipose differentiation-related protein (ADRP, also known as perilipin 2 or adipophilin), a marker of lipid droplets (25) (Figure 1D). Quantification of LipidTOX staining by flow cytometry indicated a 3-fold increase in fluorescence intensity upon activation with IFNγ, indicating a net increase in neutral lipid content rather than the mere redistribution of the existing neutral lipid pool (Figure 1E). This was supported by a 3-fold increase in TAG content in IFNγ-activated cells as measured by an enzymatic colorimetric assay in total lipid extracts (Figure 1F). Activation with IFNγ thus induces a metabolic phenotype typical of classical macrophage activation characterized by increased glycolysis, inhibition of respiration, and TAG accumulation.

### Glucose Provides Carbon to the Glycerol Headgroup of Triacyglyclerol

**Figure 2:**
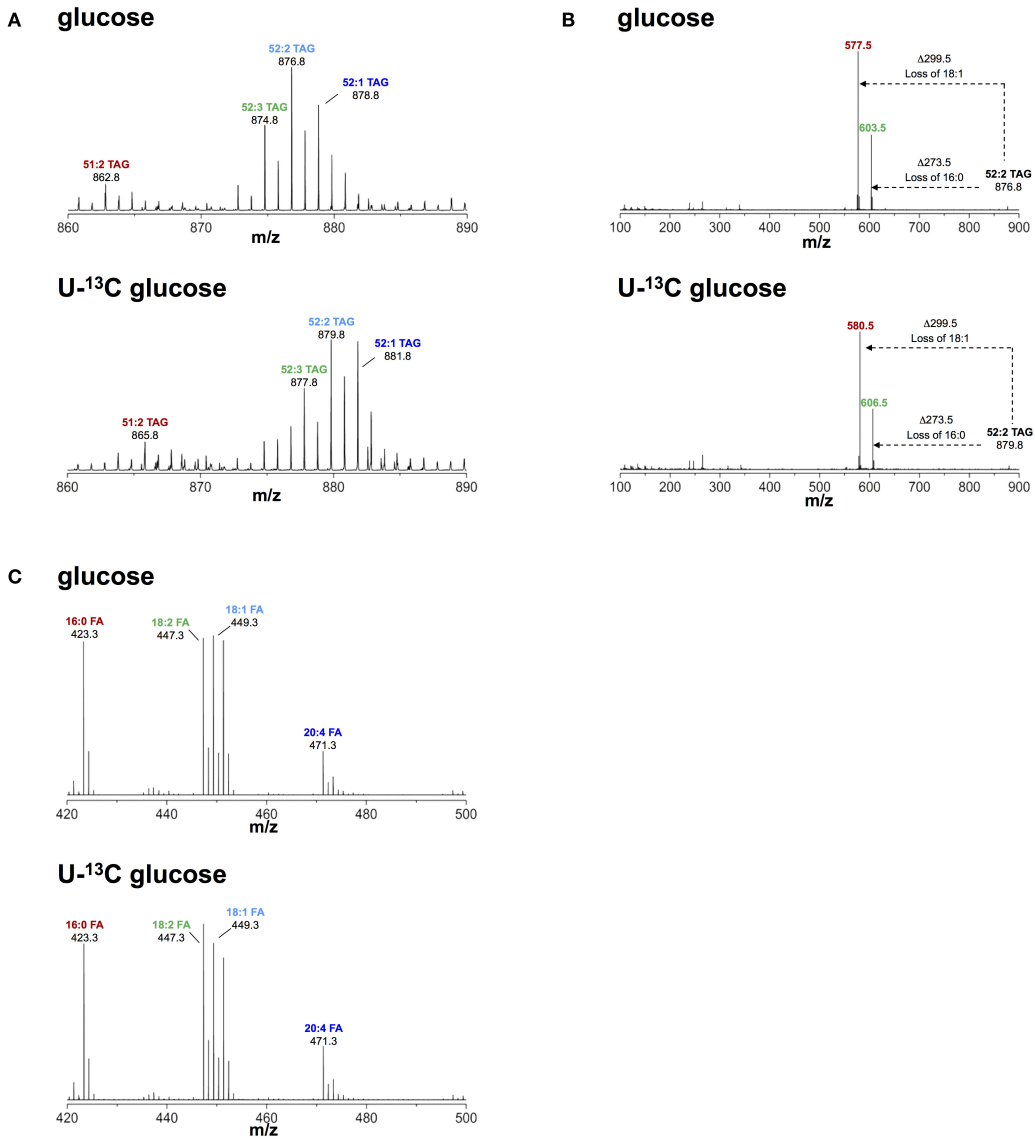
Metabolic fate of ^13^C-glucose in TAG. **(A)** ToF-MS profile of major TAG species in macrophages activated in medium containing unlabeled or U-^13^C glucose. A mass shift of 3 Da was observed in TAG species of macrophages activated in the presence of labeled glucose. **(B)** MS/MS of 52:2 TAG (m/z 876.5), a major TAG species. A mass shift of 3 Da remains after loss of fatty acid chains induced by collision fragmentation in macrophages activated in the presence of labeled glucose. **(C)** Fatty acid profile obtained from macrophages activated in medium containing unlabeled and U-^13^C glucose. There was no mass shift observed in fatty acids obtained from macrophages activated in medium containing U-^13^C glucose. Data is representative of three experiments.

Based on our observation of increased glucose uptake in activated macrophages, we questioned if glucose contributed to biosynthesis of TAG during lipid droplet formation. To this end, we activated macrophages with IFNγ in medium containing FCS, 0.8 mM glutamine, and 4.8 mM uniformly labeled (U-)^13^C glucose. We then monitored ^13^C incorporation into TAG using liquid chromatography-mass spectrometry (LC-MS). We consistently observed a mass increase of 3 Da in major TAG species extracted from cells activated in medium containing U-^13^C glucose compared to TAG from cells activated in medium containing unlabeled glucose (Figure 2A). Using collision-induced fragmentation we confirmed that this 3 Da mass increase was caused by exclusive ^13^C incorporation into the glycerol headgroup of TAG that contains three carbons (Figure 2B). Importantly, this mass shift is not compatible with *de novo* fatty acid synthesis from glucose, which would have resulted in various TAG isotopomers with mass increases in multiples of 2, as fatty acids are synthesized by the sequential addition of 2 carbon-units from acetyl-coenzyme A, and carbons contained in acetyl-coenzyme A originating from U-^13^C glucose are fully labeled (26). Similarly, we observed a mass increase of 3 Da in phosphatidylcholine (that contains one glycerol moiety) extracted from cells activated in medium containing U-^13^C glucose compared to phosphatidylcholine from cells activated in medium containing unlabeled glucose (Figure S2), indicating that glucose provides the glycerol moiety in the glycerophospholipid synthesis pathway. Moreover, the activity of cytoplasmic glycerol 3-phosphate (cG3PDH) that catalyzes the reaction dehydroxyacetone phosphate to glycerol 3-phosphate was decreased and the levels of glycerol 3-phosphate were increased 24 h after activation (Figure S3), providing further evidence of flow of glucose-derived carbon through the common TAG and glycerophospholipid synthesis pathway in macrophages activated with IFNγ. Finally, fatty acid analyses confirmed that the ^13^C label from glucose was not incorporated into the total fatty acid pool in activated cells (Figure 2C). Our results thus suggest that glucose is not a relevant substrate for *de novo* synthesis of fatty acid contained in TAG of macrophages activated with IFNγ, but instead provides glycerol to the headgroup of TAG.

### Exogenous Lipids Are Required for Neutral Lipid Accumulation Upon Activation With IFNγ

**Figure 3:**
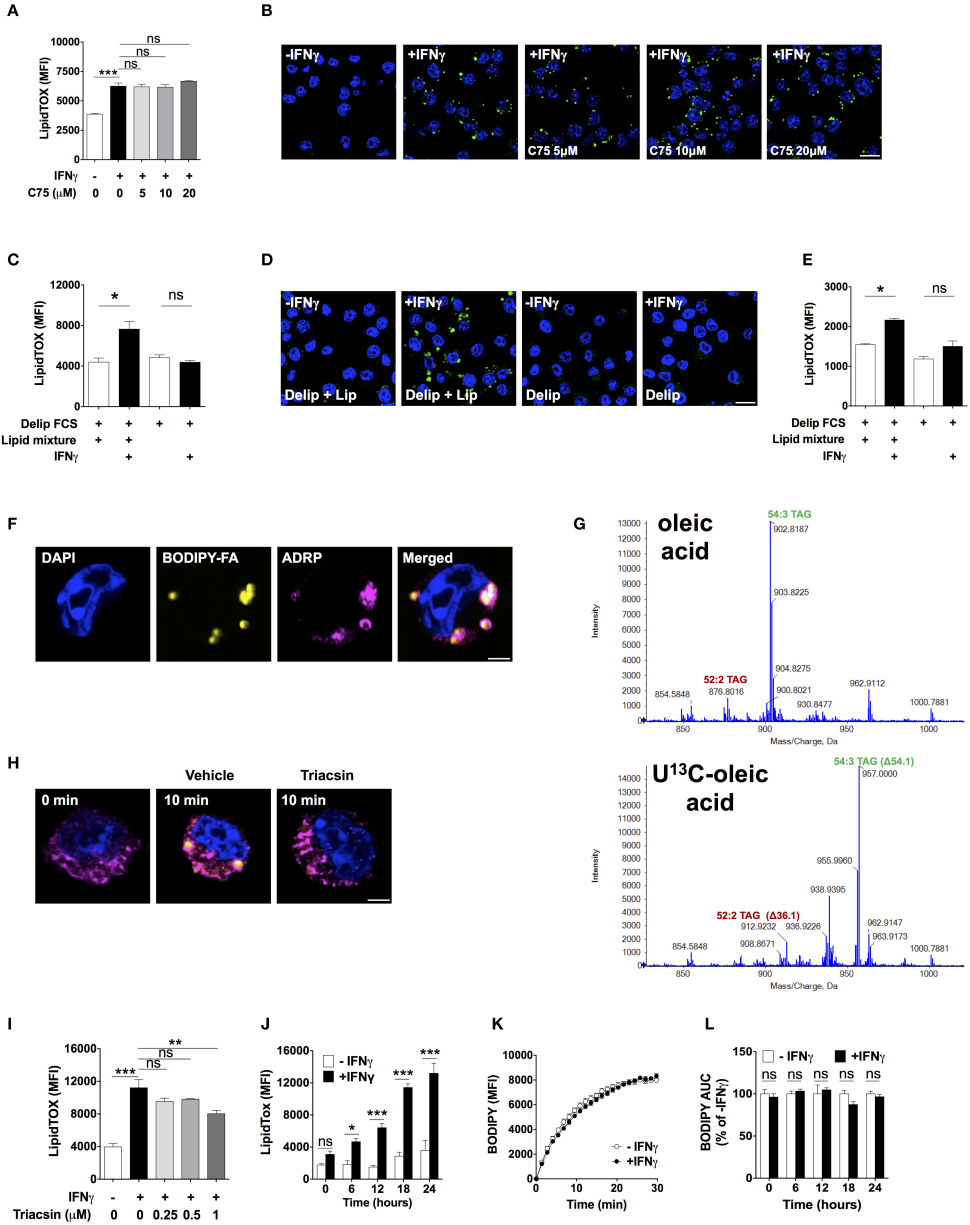
Lipid droplet accumulation induced by IFNγ is dependent on exogenous lipids not *de novo* fatty acid synthesis. **(A)** LipidTOX MFI and **(B)** confocal fluorescence image of Maf-DKO macrophages activated with IFNγ and incubated in the absence or presence of indicated concentrations of C75. Data shown as mean ± SEM of three replicates. Data is representative of three experiments. Scale bar 12.5 μm. **(C)** LipidTOX MFI and **(D)** confocal fluorescence image of Maf-DKO macrophages incubated in the presence or absence of IFNγ in medium containing delipidated (Delip) FCS, or delipidated FCS plus lipid mixture. Data shown as mean ± SEM of three replicates. Data is representative of three experiments. Scale bar 12.5 μm. **(E)** LipidTOX MFI of bone marrow-derived macrophages incubated in the presence or absence of IFNγ in medium containing delipidated (Delip) FCS, or delipidated FCS plus lipid mixture. Data shown as mean ± SEM of three replicates. Data is representative of two experiments. **(F)** Confocal fluorescence image of cells activated with IFNγ for 24 h in medium containing delipidated serum and BODIPY-fatty acid; scale bar 2.5 μm. Cells were stained with DAPI and anti-ADRP antibody. **(G)** ToF-MS profile of TAG extracted from macrophages activated in medium containing unlabeled or U-^13^C oleic acid. Note a mass shift of 36 and 54 in 52:2 TAG and 54:3 TAG, respectively, corresponding to incorporation of 2 or 3 oleic-acid chains into TAG. Data is from a single experiment. **(H)** Confocal fluorescence image of cells before and after 10 min incubation with delipidated serum and BODIPY-fatty acid. Cells were previously activated with IFNγ for 24 h followed by a 30 min incubation with triacsin (1 μM). Yellow, BODIPY-fatty acid; magenta, ADRP; scale bar 2.5 μm. Image is representative of two experiments. **(I)** LipidTOX MFI of cells activated with IFNγ in the presence of indicated concentrations of triacsin. Data shown as mean ± SEM of three replicates. Data is representative of two experiments. **(J)** LipidTOX MFI of cells incubated with or without IFNγ for different time periods. **(K)** BODIPY-fatty acid incorporation into cells measured over 30 min. **(L)** Area under the curve of BODIPY-fatty acid incorporation of cells incubated in the presence or absence of IFNγ for the indicated time periods. Data shown as mean ± SEM of five replicates. Data is representative of two experiments. ns, not significant; *p < 0.05; **p < 0.01; ***p < 0.001.

We next investigated the source of lipids that IFNγ-activated macrophages utilized to increase neutral lipid levels. To determine the role of *de novo* fatty acid synthesis, we activated macrophages in the presence of the fatty acid synthase (27) inhibitor C75 at doses previously shown to inhibit FAS activity (28–30). C75 failed to reduce LipidTOX fluorescence and did not prevent lipid droplet formation in IFNγ-activated macrophages (Figures 3A,B). Since glutamine is rapidly degraded in culture medium, it was still possible that *de novo* fatty acid synthesis from glutamine-derived carbon could play a role in TAG synthesis in the presence of higher concentrations of glutamine. We did not observe mass shifts in major TAG species extracted from cells activated in medium containing 4 mM U-^13^C, U-^15^N glutamine compared to TAG from cells activated in medium containing unlabeled glutamine (Figure S4), suggesting that glutamine is not a relevant substrate for *de novo* synthesis of fatty acid contained in TAG. As observed with low (4.8 mM) glucose concentration, there were mass shifts of 3 Da in major TAG species extracted from cells activated in medium containing 24 mM U-^13^C glucose (Figure S4). Together, these data further support the notion that *de novo* fatty acid synthesis was not required for neutral lipid accumulation.

We next tested the role of exogenous lipids by activating macrophages in delipidated serum in the presence or absence of a lipid mixture containing fatty acids and cholesterol. Addition of lipid mixture to delipidated serum, but not delipidated serum alone, induced lipid droplet formation and increased LipidTOX levels in IFNγ-activated cells (Figures 3C,D). We tested whether this observation was applicable to other macrophage sources other than Maf-DKO cells. Indeed, activation of bone marrow-derived macrophages with IFNγ induced lipid accumulation that was also dependent on external lipids (Figure 3E).

To trace the fate of external lipids, we activated cells with IFNγ in the presence of delipidated serum supplemented with a 12-carbon long fatty acid analog labeled with the fluorescent dye BODIPY (henceforth BODIPY-fatty acid). BODIPY-fatty acid accumulated within lipid droplets, suggesting that exogenous fatty acid incorporated into TAG contained in these organelles (Figure 3F). We confirmed this by activating cells in the presence of U-^13^C oleic acid. This resulted in TAG mass increases in multiples of 18 as determined by LC-MS, indicating incorporating one or several U-^13^C oleic acid chains into TAG (Figure 3G).

Externally derived fatty acids must be activated before incorporation into TAG by esterification with coenzyme A through a reaction catalyzed by fatty acyl-CoA synthetase. Indeed, the fatty acid CoA synthetase inhibitor triacsin prevented BODIPY-fatty acid accumulation in lipid droplets (Figure 3H), reduced LipidTOX fluorescence intensity (Figure 3I), and prevented lipid droplet formation (Figure S5), indicating that fatty acid esterification with coenzyme A is needed for fatty acid incorporation into lipid droplets in activated macrophages. Together, these data demonstrate that neutral lipids accumulating in activated macrophages originate from glucose-derived glycerol as well as directly incorporated externally-derived fatty acids, with no need for *de novo* fatty acid synthesis.

### Inhibition of Mitochondrial Respiration by iNOS-Derived Nitric Oxide Induces Neutral Lipid Accumulation

Lipid droplets might have accumulated because of increased fatty acid uptake. We observed increased expression of the scavenger receptor CD36 upon activation with IFNγ, but addition of a blocking anti-CD36 antibody previously shown to inhibit fatty acid uptake (31) did not diminish the LipidTOX increase induced by IFNγ (Figure S6). In fact, despite continuously increasing LipidTOX fluorescence over a period of 24 h in activated macrophages (Figure 3J), we did not observe differences in BODIPY-fatty acid incorporation rate between activated and non-activated macrophages at any time point studied (Figures 3K,L). Thus, neutral lipid accumulation in IFNγ-activated macrophages is not accounted for by increased uptake of fatty acid. Instead, the fate of internalized fatty acids might differ between activated and non-activated macrophages.

To gain additional insight into the underlying metabolic program required for lipid droplet accumulation we used quantitative proteomics. We identified 2,865 proteins, out of which 98 were differentially abundant (63 up, 35 down; FDR *q* < 0.1, fold change > 2; Table S1). Proteins pertaining to the electron transport chain complexes I and II were down-regulated (2- to 2.9-fold change; Table S2; Figure S7). In order to detect small but coordinated changes in the expression of proteins belonging to predefined pathways, we used the gene set enrichment analysis (GSEA) algorithm (32) on our proteomics dataset. Results showed coordinated up- or downward changes in the expression of proteins belonging to 12 pathways (FDR *q* < 0.05) (Table S3). The pathways with the two highest enrichment scores associated with proteins up-regulated by IFNγ were the “Proteasome” (normalized enrichment score, NES 2.159) and the “Antigen and presentation” (NES 2.139) pathways, further supporting a normal response of Maf-DKO macrophages to IFNγ. Metabolic pathways associated with IFNγ up- or down-regulated proteins were the “Glycolysis/gluconeogenesis” pathway (NES 1.719), and the “Oxidative phosphorylation” pathway (NES −1.792), respectively (Figures S8A,B), consistent with our results of increased glycolysis and decreased OCR. The coordinated down-regulation of proteins pertaining to oxidative phosphorylation observed in our proteomics dataset, together with published research that inhibition of mitochondrial β-oxidation can lead to lipid droplet accumulation under a continued supply of fatty acid (33–35), hinted at impaired mitochondrial respiration as the underlying mechanism for accumulation of neutral lipids in macrophages activated with IFNγ.

**Figure 4:**
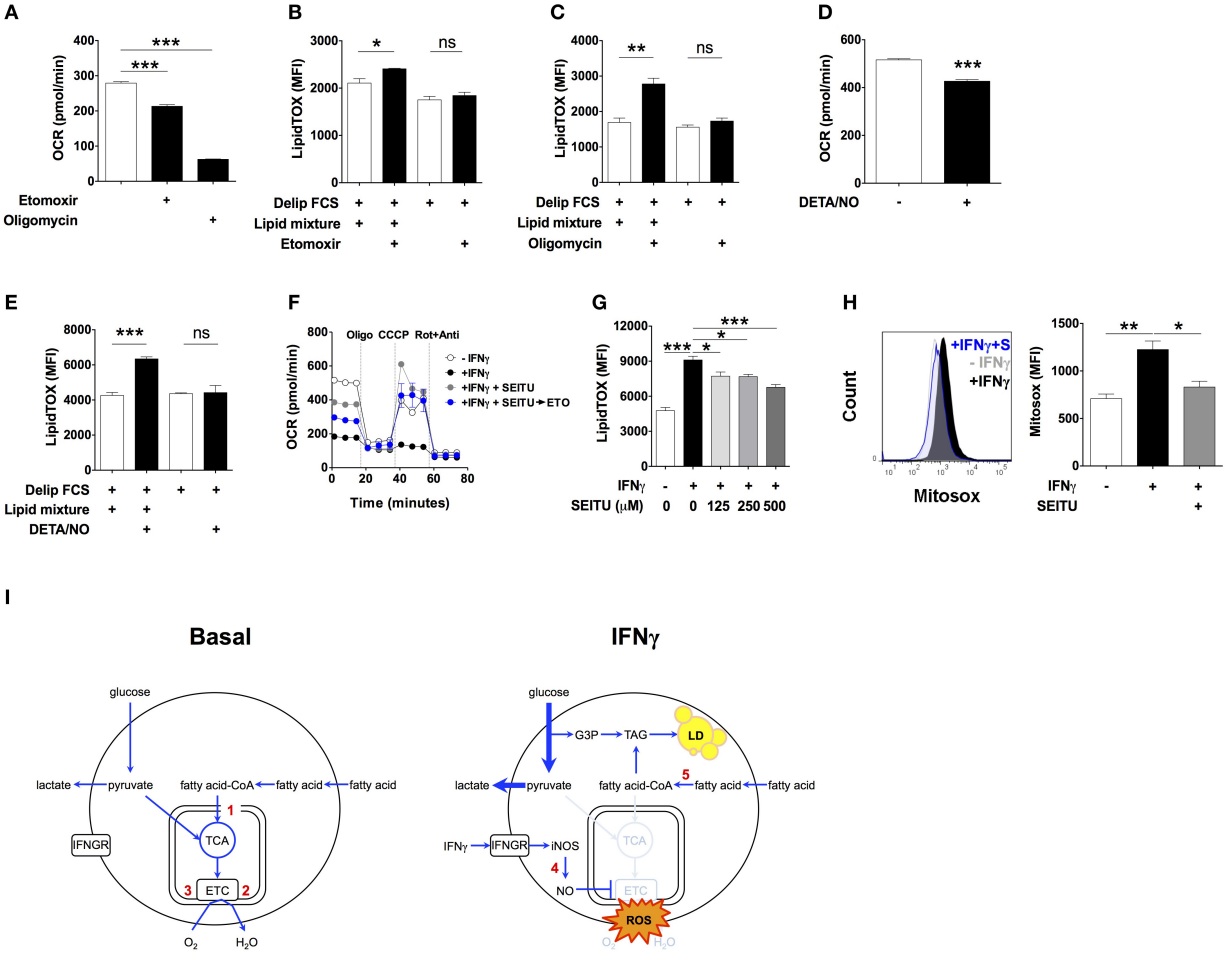
Inhibition of mitochondrial respiration by iNOS induces lipid droplet accumulation. **(A)** Maf-DKO macrophages were incubated for 24 h in medium containing FCS plus or minus etomoxir (200 μM) or oligomycin (10 μM). Oxygen consumption rate (OCR) was then measured in the presence of drug. Three consecutive basal OCR measurements were averaged. Data shown as mean ± SEM of triplicates, and is representative of two experiments. **(B,C)** LipidTOX MFI of cells incubated in medium with delipidated FCS plus or minus lipid mixture, with or without etomoxir (200 μM) or oligomycin (10 μM). Data shown as mean ± SEM of 3 replicates. Data is representative of two experiments. **(D)** OCR of cells incubated for 24 h without or with IFNγ plus DETA/NO (100 μM). Three consecutive basal OCR measurements were averaged. Data shown as mean ± SEM of triplicates, and is representative of two experiments. **(E)** LipidTOX MFI levels in cells incubated in medium containing delipidated FCS plus or minus lipid mixture, with or without DETA/NO (100 μM). Data shown as mean ± SEM of triplicates, and is representative of two experiments. **(F)** Cells were incubated for 24 h without or with IFNγ plus SEITU (500 μM). OCR was then measured before and after sequential addition of oligomycin, CCCP and rotenone plus antimycin. The blue trace shows OCR of cells in which etomoxir (200 μM) was added after 24 h of incubation with IFNγ plus SEITU, and 30 min before measurements started to be obtained. **(G)** LipidTOX MFI levels in cells incubated in FCS without or with IFNγ plus indicated concentrations of SEITU. Data shown as mean ± SEM of triplicates, and is representative of two experiments. **(H)** Histogram and quantification of Mitosox MFI in Maf-DKO cells incubated for 24 h with or without IFNγ or with IFNγ plus SEITU (S) (500 μM). Data shown as mean ± SEM of triplicates, and is representative of two experiments. ns, not significant *p < 0.05; **p < 0.01; ***p < 0.001. **(I)** Model of metabolic changes leading to TAG accumulation in activated macrophages. Macrophages oxidize fatty acid under basal conditions. Upon activation with IFNγ, nitric oxide produced by INOS inhibits mitochondrial respiration, and as a consequence, β-oxidation. External fatty acid that would otherwise be oxidized in mitochondria is esterified with glycerol 3-phosphate originating from glucose leading to synthesis of TAG and its accumulation in lipid droplets. Numbers in red indicate the inhibition site of the following compounds: 1, etomoxir: inhibits carnitine palmitoyltransferase-1; 2, oligomycin: inhibits ATP synthase; 3, DETA/NO: inhibits respiratory complexes I and IV; 4, SEITU: inhibits iNOS; 5, triacsin: inhibits fatty acyl-CoA synthetase.

To test this possibility, we first determined macrophage β-oxidation activities under basal conditions. Incubation of macrophages for 24 h with the carnitine palmitoyltransferase I inhibitor etomoxir reduced OCR by 23.5 ± 0.4% (Figure 4A), which is equivalent to 65.7 ± 4.8 pmoles per minute per million cells (Table S4). This accounts for 30.3 ± 0.6% of the oligomycin-sensitive OCR, indicating that mitochondrial fatty acid oxidation accounts for 30.3% of the oxygen consumption coupled to ATP production (Figure 4A). Based on our calculations (Table S4), the estimated amount of fatty acid oxidized in mitochondria in 24 h by non-activated macrophages corresponds to 11.7 times the amount of TAG accumulated by macrophages activated with IFNγ over the same period of time, assuming total incorporation of fatty acid exclusively into TAG. This relatively high level of β-oxidation under basal conditions suggested that even partial inhibition of mitochondrial fatty acid oxidation could fully account for the accumulation of TAG observed in macrophages activated with IFNγ. In line with this, etomoxir induced a small but statistically significant increase in LipidTOX fluorescence intensity in non-activated cells incubated in medium containing delipidated serum supplemented with lipid mixture (Figure 4B). Importantly, etomoxir failed to increase LipidTOX fluorescence intensity and to induce lipid droplet formation in cells incubated without lipid mixture (Figure 4B; Figure S9A). Moreover, inhibition of mitochondrial respiration with the ATP synthase inhibitor oligomycin also increased LipidTOX fluorescence intensity and induced lipid droplet formation in non-activated cells (Figure 4C; Figure S9B). As with etomoxir, the increase in LipidTOX fluorescence intensity and lipid droplet accumulation induced by oligomycin was dependent on exogenous lipids (Figure 4C; Figure S9B). Taken together, these findings indicate that macrophages oxidize fatty acid under basal conditions, and that inhibition of fatty acid oxidation or mitochondrial respiration is sufficient to increase neutral lipid content in non-activated macrophages, provided exogenous lipids are available.

Next, we tested if inhibition of mitochondrial respiration by nitric oxide contributed to accumulation of neutral lipid upon activation with IFNγ (36, 37). We incubated non-activated macrophages with the nitric oxide donor molecule diethylenetriamine/NO adduct (DETA/NO) for 24 h (38, 39). DETA/NO significantly decreased OCR (Figure 4D), increased LipidTOX fluorescence intensity and induced lipid droplets in cells incubated in medium containing delipidated serum complemented with lipid mixture, but not without (Figure 4E; Figure S9C). Conversely, SEITU, a selective iNOS inhibitor, partially reversed the OCR inhibition induced by IFNγ and completely recovered mitochondrial spare respiratory capacity (Figure 4F). SEITU also induced a partial but significant dose-dependent reduction in LipidTOX fluorescence intensity and lipid droplets (Figure 4G; Figure S10). Notably, etomoxir reduced OCR even when added to cells after 24 h of incubation with IFNγ plus SEITU (Figure 4F), indicating that SEITU preserved the capacity of mitochondria to perform β-oxidation, even in macrophages activated with IFNγ. This finding further supports the notion that neutral lipids accumulate due to reduced mitochondrial oxidation of fatty acid. Activation with IFNγ also increased mitochondrial reactive oxygen species (mROS) levels, an indicator of mitochondrial damage (40). Macrophage activation in the presence of SEITU significantly reduced mROS levels (Figure 4H), suggesting that SEITU inhibited neutral lipid accumulation by preventing mitochondrial damage and thus preserving fatty acid oxidation in mitochondria. Altogether these data indicate that iNOS-derived nitric oxide inhibits mitochondrial respiration, and as a consequence, fatty acid oxidation, leading to neutral lipid accumulation that is dependent on exogenous lipids.

## Discussion

Inspired by lipid metabolism of cancer cells, the current model of classical M1 activation of macrophages assumes a switch from fatty acid degradation to *de novo* fatty acid synthesis. In order to study the contribution of metabolites to lipid synthesis, previous studies have relied on the detection of radioactivity in total lipids extracted from cells incubated in the presence of ^14^C-labeled glucose (11, 17, 41, 42). Based on these data, it was proposed that fatty acid is synthesized *de novo* from glucose-derived carbon in classically-activated macrophages and dendritic cells (11, 17). However, this approach is non-informative about the site of carbon incorporation, i.e., lipid headgroup vs. fatty acid, and thus no proof for fatty acid biogenesis. In the present study, we revisited this issue using current lipidomics techniques. We could confirm incorporation of carbon derived from glucose into macrophage lipids but, surprisingly, this was exclusively restricted to the glycerol moiety in the headgroup of TAG, but not fatty acids. The lack of any impact of blocking FAS by C75 confirmed no need for fatty acid *de novo* synthesis. It remains possible however that glucose contributes carbon for synthesis of other lipid species, for example through citrate in the mevalonate pathway. Instead, carbon atoms in the acyl chains of TAG derive largely from exogenous fatty acids, and neutral lipid accumulation is indeed dependent on exogenous lipids and their activation by esterification with coenzyme A.

In non-activated macrophages, exogenous lipids are taken up at similar rates compared to activated cells. However, non-activated macrophages degrade lipids by β-oxidation, whereas β-oxidation is blocked in activated macrophages. This is in part due to generation of iNOS-derived nitric oxide, which can directly or through reactive nitrogen species inhibit oxidative phosphorylation through S-nitrosylation of protein cysteine residues of respiratory complexes I and IV (43, 44). Inhibition of oxidative phosphorylation could also be accounted for by itaconate-induced inhibition of SDH, a mechanism known to occur in activated macrophages (45). In addition, our proteome dataset indicates copy number reduction of multiple proteins involved in the mitochondrial respiratory chain, suggestive of loss of mitochondrial mass. Such reduction of mitochondrial content can be caused by mitophagy, the selective degradation of damaged mitochondria through autophagy. Indeed, IFNγ induces autophagy in macrophages (46), and our own results show that IFNγ increases mROS levels, both indications for damaged mitochondria (47, 48). It is this block in respiration that prevented degradation of fatty acids by β-oxidation. This diminished fatty acid catabolism, but not *de novo* biogenesis of fatty acids, is the basis of lipid accumulation in macrophages activated with IFNγ (Figure 4I).

Increased glucose uptake is a hallmark of macrophage pro-inflammatory activation. High glucose uptake supports phagocyte function by maintaining high ATP synthesis rates through glycolysis, and provides reduction potential through the pentose phosphate pathway (21, 49). We show now that glucose also contributes carbon specifically to the glycerol headgroup of TAG. In the glycerolipid synthesis pathway, the glycolytic intermediate dihydroxyacetone phosphate is reduced in the reaction catalyzed by cytoplasmic glycerol 3-phosphate dehydrogenase producing glycerol 3-phosphate. Glycerol 3-phosphate is then acylated by glycerol 3-phosphate acyltransferase, the rate limiting step of *de novo* TAG synthesis (50). We propose that increased glycolysis rate also serves to supply glycerol 3-phosphate for glycerolipid synthesis in activated macrophages. This could serve the function of decreasing levels of otherwise toxic fatty acids, and/or of providing lipids to endoplasmic reticulum and Golgi apparatus required for increased cytokine production, for example (11, 51, 52).

In our study, macrophage activation underwent in medium containing glucose and glutamine at physiological concentrations. Nonetheless, lipids in tissue culture medium do not necessarily represent qualitatively nor quantitatively the lipid composition of plasma *in vivo*. This is particularly the case in sterile and non-sterile systemic inflammation, conditions characterized by abnormal plasma levels of lipoprotein and TAG (53, 54). In contrast to our findings, previous studies have failed to show increase in glucose uptake in resident peritoneal macrophages activated with IFNγ plus TNF (55). This discrepancy can be explained by functional differences between macrophage sources including the magnitude of their responses to pro-inflammatory and anti-inflammatory stimuli (56, 57). Macrophages can also be activated by other stimuli including pathogen-associated molecular patterns (PAMPs) such as LPS. It may be interesting to investigate in future studies if similar metabolic activities drive lipid accumulation in activated macrophages also under these conditions.

In conclusion, our findings establish a new metabolic pathway in activated macrophages in which exogenous fatty acids are the primary source of acyl chains that are then esterified with *de novo* synthesized glycerol from glucose yielding TAG. In comparison to the previously assumed *de novo* fatty acid biosynthesis, this pathway is a more efficient way to store lipids since it requires minimal energy and overall metabolic activities. This lipogenesis pathway is fundamentally different from that in cancer cells that synthesize their own fatty acids from glutamine and glucose, and highlights that metabolism of activated macrophages and cancer cells might be more different than previously assumed.

## Materials and Methods

### Cell Culture

MafB/c-Maf double deficient (Maf-DKO) macrophages were a kind gift from Dr. Michael H. Sieweke (Center d'Immunologie de Marseille-Luminy). Maf-DKO cells were grown in DMEM containing 20% L929-conditioned medium and 10% FCS. BMDM were differentiated from bone marrow cells obtained from C57BL6 mice in the presence of DMEM containing 20% L929-conditioned medium and 10% FCS. Maf-DKO cells were predominantly octaploid (8c) whereas BMDM were diploid (2c) and octaploid (8c) as determined by DNA quantification with DAPI staining (data not shown). For experiments, cells were incubated for 24 h at 37°C and 5% CO_2_ in low glucose, low glutamine medium (henceforth LGLG medium) which contained DMEM, 44 mM sodium bicarbonate, 10% FCS, glucose (4.8 mM), and glutamine (0.8 mM) with or without IFNγ 10 ng/mL or IL-4 10 ng/mL (both from Preprotec), and C75, etomoxir, triacsin, oligomycin, DETA/NO, or SEITU (all from Sigma) at the indicated concentrations. In a set of experiments, FCS was replaced with delipidated serum (Lipoprotein deficient serum from fetal calf, Sigma) plus a 1:100 dilution of lipid mixture (Lipid mixture 1, Sigma) containing cholesterol, and arachidonic, linoleic, linolenic, myristic, oleic, palmitic, and stearic acids.

### Cell Surface Markers

Cells were incubated in 24-well plates for 24 h after which they were washed once with cold PBS and detached with cold PBS/EDTA 1 mM. Cells were stained with anti-CD86 (BD, clone GL1), I-A/I-E (BD, clone 2G9) or anti-CD36 (Merck, clone 63) antibodies in FACS buffer (PBS, EDTA 2 mM, 2% FCS) for 30 min on ice, washed once, and resuspended for cytometry analysis. Cells were acquired with an LSRFortessa II flow cytometer (BD Biosciences) and analyzed with FlowJo (Tree Star, Inc.).

### Neutral Lipid Staining

Cells were incubated in 24-well plates for 24 h after which they were washed once with cold PBS, fixed with PBS containing formaldehyde 4% for 10 min, and washed three times with PBS. Cells were incubated at room temperature (RT) for 30 min with HCS LipidTOX Green Neutral Lipid (ThermoFisher Scientific) diluted 1:1,000 in PBS, washed three times with PBS, scraped off, and resuspended in PBS for flow cytometry analysis. For microscopy analysis, cells were grown on microscope slides inside wells of 24-well plates followed by the same procedure as for flow cytometry with the addition of DAPI. For ADRP staining, cells were incubated at RT for 1 h in blocking buffer (PBS, 0.2% TritonX-100, 5% goat serum). Then, rabbit polyclonal anti-ADRP antibody (Abcam) was added at a final 1:200 dilution, and cells were incubated for 2 h at RT. After washing with blocking medium, cells were incubated at RT for 2 h in blocking medium containing Alexa Fluor 647-conjugated goat anti-rabbit antibody (ThermoFisher Scientific) at a 1:1,000 dilution. After washing with blocking medium, cells were mounted with Fluoromount-G (Southern Biotech). Images were taken with a Leica SP8 confocal microscope, and analyzed with Fiji.

### Mitochondrial Reactive Oxygen Species

Cells were washed once with cold PBS. PBS was then removed and cold PBS/EDTA 5 μM solution was added. Cells were incubated for 5 min at RT, gently detached by pipetting, and transferred to FACS tubes and kept on ice. Tubes were spun down at 200 g for 5 min at 4°C, after which PBS/EDTA was removed. Cells were resuspended in 200 μL of LGLG medium containing 2 μL of a 1:10 dilution of Mitosox (ThermoFisher Scientific), and incubated at 37°C in a water bath for exactly 25 min. DAPI was then added to exclude dead cells and cells were analyzed by flow cytometry.

### Metabolite and Cytokine Quantification

Glucose, lactate, and triacylglycerol were determined using commercially available enzymatic assay kits (BioAssay Systems or BioVision) following the manufacturers' instructions. Glycerol 3-phosphate and activity of cG3PDH were quantified as previously described (58, 59). Cytokines were measured using the Cytometric Bead Array Flex Set system (BD Biosciences) and analyzed with FlowJo (Tree Star, Inc.). Cytokine detection limits are IL-1β: 1.9 pg/mL; IL-6: 1.4 pg/mL; IL-10: 9.6 pg/mL; and TNF-α: 2.8 pg/mL.

### Western Blot

Total cell protein was extracted from cells using CelLytic M (Sigma) and kept at −20°C until further analysis. Protein was heated for 5 min at 50°C (for mitochondrial respiratory complexes) or 95°C (for iNOS and arginase-1) in 5x Laemmli buffer containing 2-mercaptoethanol. Samples were loaded into acrylamide gels and protein was transferred to PVDF membranes followed by detection with chemiluminiscence using the total OXPHOS rodent WB antibody cocktail (Abcam), or anti-INOS (Abcam, ab15323), anti-arginase-1 (Santa Cruz, sc-18354) or anti-β-actin antibodies (Rockland Immunochemicals) plus appropriate HRP-conjugated secondary antibodies.

### Oxygen Consumption Measurements

Oxygen consumption rate was measured with an XF^e^ Extracellular Flux Analyzer (Seahorse Bioscience). Cells were incubated for 24 h in XF-96 cell culture plates at a density of 10^5^ cells/200 μL per well in LGLG medium in the presence or absence of IFNγ 10 ng/mL, and SEITU, etomoxir, DETA-NO and oligomycin at the indicated concentrations. One hour prior to the experiment, LGLG medium was removed and 175 μL of assay medium (LGLG medium containing 44 mM sodium chloride instead of 44 mM sodium bicarbonate in order to prevent pH buffering and to maintain medium osmolarity) were added. Drugs (25 μL) were injected during the assay at the following final concentrations: oligomycin (1 μM), CCCP (carbonyl cyanide m-chloro phenyl hydrazone) as uncoupler (2 μM), and rotenone (100 nM) plus antimycin (1 μM). The experiment was performed at 37°C and 20% oxygen using a mix-wait-measure protocol of 3-0-3 min with three initial basal rate measurements.

### Fatty Acid Uptake

Fatty acid uptake was determined by measuring incorporation of the fluorescent fatty acid analog C_1_-BODIPY 500/510-C_12_ (Life Technologies) into cells using a fluorescent plate reader, as previously described (60). Briefly, cells were cultured for 24 h in LGLG medium in 96-well black plates with clear flat bottom wells at a concentration of 10^5^ cells per well in the presence or absence of IFNγ 10 ng/mL. Cells were washed once with warm PBS and incubated at 37°C for 30 min with 100 μL of LGLG without FCS (serum free LGLG medium). Staining solution consisted of one part 2x serum free LGLG medium plus one part 8% trypan blue that served as fluorescence quencher. To this staining solution C_1_-BODIPY 500/510-C_12_ diluted 1:4,000 from a 1 mg/mL stock was added. Fatty acid incorporation was started by adding 100 μL of pre-warmed (37°C) staining solution (final C_1_-BODIPY 500/510-C_12_ concentration 309 nM) to each well already containing 100 μL of serum free LGLG. Fluorescence (Ex485/Em528) was measured from the bottom of the plate on a Synergy H4 (BioTek) plate reader set at 37°C. Data were acquired at intervals of 80 s for up to 30 min, time at which fluorescence plateaued. In a different experiment, cells were incubated for 24 h in LGLG medium containing delipidated serum, IFNγ 10 ng/mL and C_1_-BODIPY 500/510-C_12_ at a final dilution of 1:4,000. In another set of experiments, cells were incubated for 24 h in LGLG medium containing FCS and IFNγ 10 ng/mL. Cells were washed once with warm PBS, and incubated at 37°C for 30 min in LGLG medium containing delipidated serum plus triacsin (1 μM), after which C_1_-BODIPY 500/510-C_12_ was added. After 10 min of incubation at 37°C cells were washed swith PBS, fixed, and stained with DAPI and anti-ADRP antibody as mentioned above.

### Mass Spectrometry Analyses of Lipids With ^13^C-Labeled Compounds

Cells were incubated in 6-well plates in the presence of IFNγ 10 ng/mL in LGLG medium. Some wells contained 4.8 mM uniformly-labeled (U-^13^C) glucose (Cambridge Isotope Laboratories) instead of glucose. In another set of experiments, cells were incubated in the presence of IFNγ 10 ng/mL in medium containing 24 mM uniformly-labeled (U-^13^C) glucose plus L-glutamine 4, or 24 mM of glucose plus 4 mM U-^13^C, U-^15^N L-glutamine (Cambridge Isotope Laboratories). In another set of experiments, cells were incubated in the presence of LGLG containing delipidated serum instead of FCS plus 50 μM oleic acid or U-^13^C oleic acid (Sigma) conjugated to BSA. After 24 h, cells were washed once with cold PBS, scraped off, counted, resuspended in 100 μL of PBS, and kept frozen at −80°C until further analysis. For lipid extraction, cells were thawed and extracted using modified Bligh and Dyer method (61). Samples were kept frozen at −80°C until further analysis. For fatty acid analyses, lipids were hydrolyzed and derivatized using the AMP+ mass spectrometry kit (Cayman) according to the manufacturer's instructions. TAG analyses were performed using a SCIEX tripleToF mass spectrometer (6600) coupled to an Agilent liquid chromatography (1290) system. Separation of TAG was achieved using a C18 column as previously described (62). For fatty acid analysis, the derivatized fatty acids were directly infused into the mass spectrometer and analyzed using ToF-MS scan.

### Proteomics

Cells were incubated in the presence or absence of IFNγ for 24 h, after which they were washed three times with cold PBS. They were then scrapped off, counted, spun down, resuspended in PBS, and kept frozen at −80°C until further quantitative proteomic analysis as described previously (63). In brief, proteins were extracted, reduced, alkylated, and digested using LysC and trypsin. After desalting, peptide samples were analyzed by nanoscale liquid chromatography-tandem mass spectrometry (nLC-MS/MS) and identified by database searching against all predicted proteins for *Mus musculus* downloaded from SwissProt (2016/10/05). Proteins were quantified by MS1-based label-free quantification and statistical analysis was performed using SafeQuant (63). GSEA analysis (32) was performed using 1,000 sample permutations. Protein sets obtained from the KEGG pathway database were tested for enrichment within our proteome data set ranked according to the signal-to-noise metric. Pathways identified in this way with a FDR *q* < 0.05 were considered as discoveries.

### Statistical Analysis

Comparisons between two groups were made using a two-tailed unpaired *t*-test. Comparisons between three or more groups were made using a one-way analysis of variance with Bonferroni correction. *P* < 0.05 were considered statistically significant. Analyses were performed with Prism 5 (GraphPad).

## Data Availability Statement

The mass spectrometry proteomics data have been deposited to the ProteomeXchange Consortium via the PRIDE partner repository with the dataset identifier PXD017148 and 10.6019/PXD017148. Other raw data supporting the conclusions of this article will be made available by the authors, without undue reservation, to any qualified researcher.

## Author Contributions

MR-B conceived the study. MR-B, DB, and XG designed experiments. XG, AS, and MR-B performed experiments and analyzed data. XG, AS, and DB provided resources. MR-B and XG wrote the paper.

### Conflict of Interest

The authors declare that the research was conducted in the absence of any commercial or financial relationships that could be construed as a potential conflict of interest.
