# Supplementary material for: Classical Activation of Macrophages Leads to Lipid Droplet Formation Without de novo Fatty Acid Synthesis
Source: Front Immunol. 2020 Feb 18;11:131. doi: 10.3389/fimmu.2020.00131 (PMC7040478; doi:10.3389/fimmu.2020.00131)
Supplement: Supplementary file 1 [file Data_Sheet_1.PDF]

Classical activation of macrophages leads to lipid droplet formation without de novo fatty acid synthesis

Xue Li Guan[1], Alexander Schmidt[2], Dirk Bumann[3*], and Mauricio Rosas-Ballina[3*]

[1] Lee Kong Chian School of Medicine, Nanyang Technological University, Singapore. [2] Proteomics Core Facility, Biozentrum, University of Basel, 4056 Basel, Switzerland. [3] Focal Area Infection Biology, Biozentrum, University of Basel, 4056 Basel, Switzerland.

Correspondence:

Mauricio Rosas-Ballina
morsba@yahoo.com

Dirk Bumann
dirk.bumann@unibas.ch

Supplementary Figures

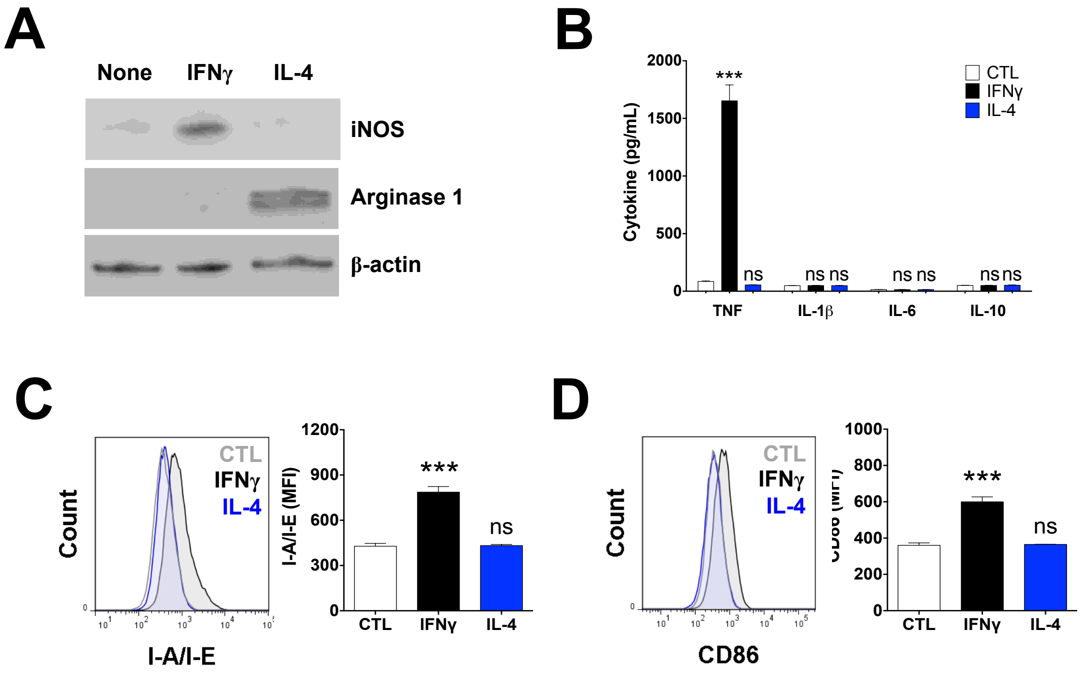

Supplementary Figure 1. Activation of Maf-DKO cells with IFNγ or IL-4 leads to M1 and M2 polarization. Maf-DKO macrophages were incubated in the presence or absence of IFNγ or IL-4 for 24 hours. **(A)** Immunoblot of iNOS and arginase-1. Data obtained from a single experiment. **(B)** Cytokine levels in supernatants. Data shown as mean ± SEM of 3 replicates. Data obtained from a single experiment. Histograms of cells stained for **(C)** I-A/I-E and **(D)** CD86. Data shown as mean ± SEM of 3 replicates. Data is representative of 2 experiments. ns, not significant; *** $p < 0.001$ compared to control.

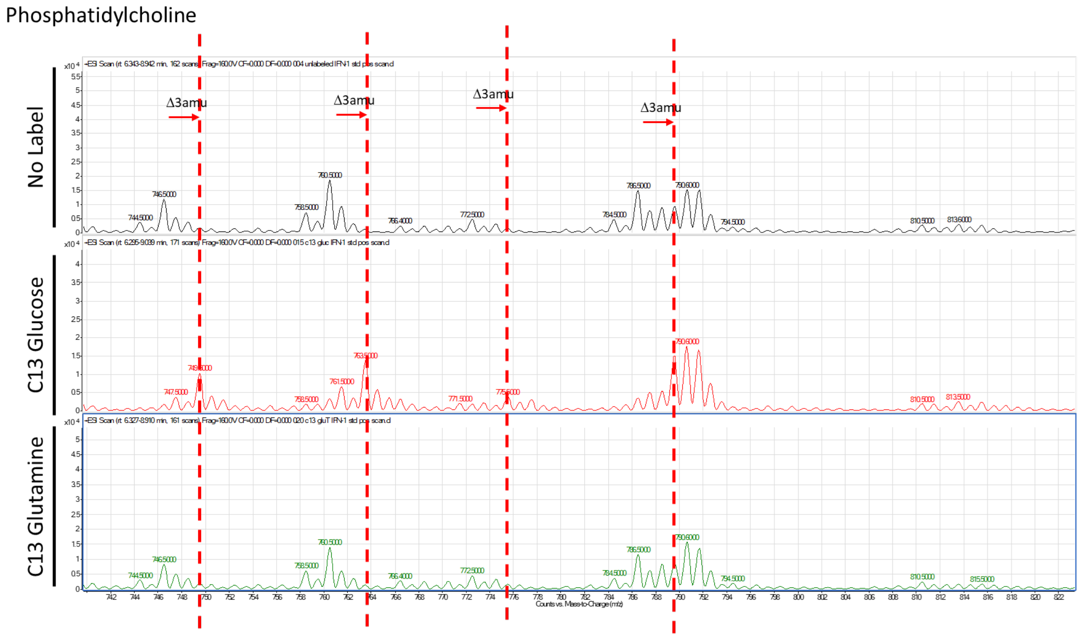

Supplementary Figure 2. Metabolic fate of U-^{13}C glucose and U-^{13}C, U-^{15}N L-glutamine in phosphatidylcholine. ToF-MS profile of major phosphatidylcholine species in macrophages activated in medium containing unlabeled 24 mM glucose and 4 mM glutamine (No Label), 24 mM U-^{13}C glucose plus 4 mM L-glutamine (C13 Glucose), or 24 mM glucose plus 4 mM U-^{13}C, U-^{15}N L-glutamine (C13 Glutamine). A mass shift of 3 Da was observed in phosphatidylcholine species of macrophages activated in the presence of labeled glucose but not in the presence of labeled glutamine. Data is representative of 2 experiments.

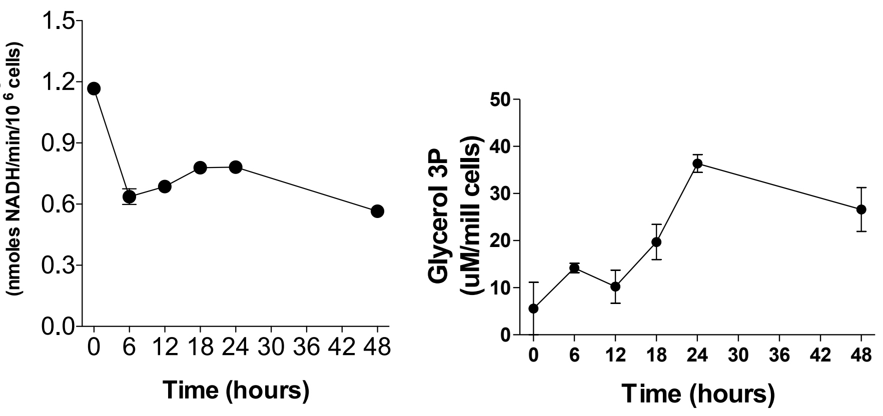

Supplementary Figure 3. IFNγ down-regulates glycerol 3-phosphate dehydrogenase activity and increases the glycerol 3-phosphate pool. Samples for cytoplasmic glycerol 3-phosphate dehydrogenase (cG3PDH) activity (left) and glycerol 3-phosphate content (right) were obtained before and at specified time points after addition of IFNγ. Data obtained from a single experiment.

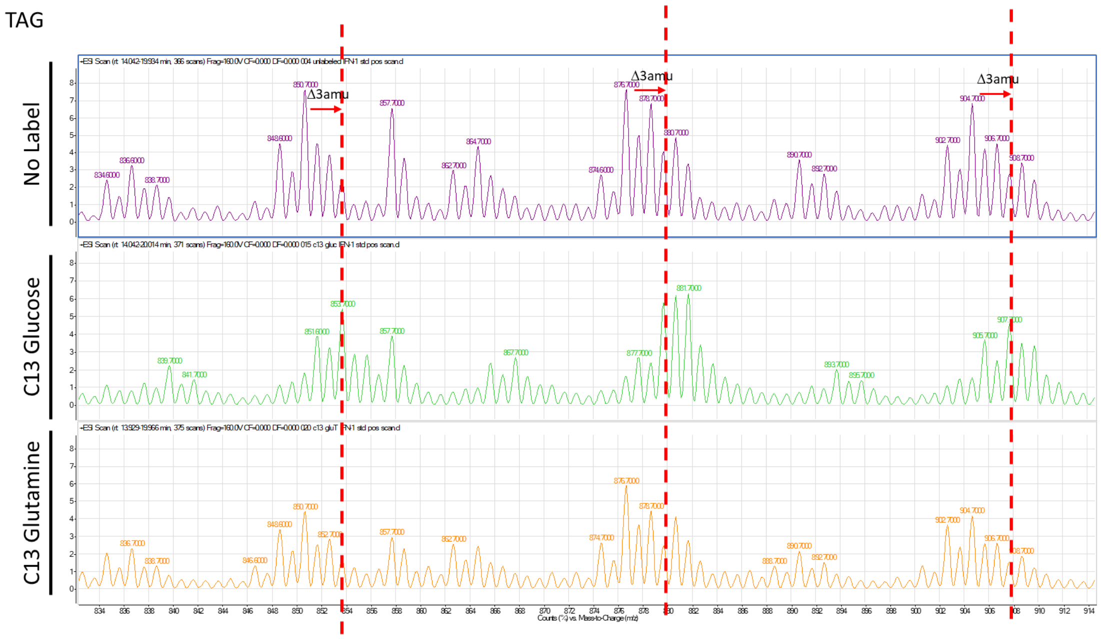

Supplementary Figure 4. Metabolic fate of U-^{13}C glucose and U-^{13}C, U-^{15}N L-glutamine in TAG. ToF-MS profile of major TAG species in macrophages activated in medium containing unlabeled 24 mM glucose and 4 mM glutamine (No Label), 24 mM U-^{13}C glucose plus 4 mM L-glutamine (C13 Glucose), or 24 mM glucose plus 4 mM U-^{13}C, U-^{15}N L-glutamine (C13 Glutamine). A mass shift of 3 Da was observed in TAG species of macrophages activated in the presence of labeled glucose but not in the presence of labeled glutamine. Data is representative of 2 experiments.

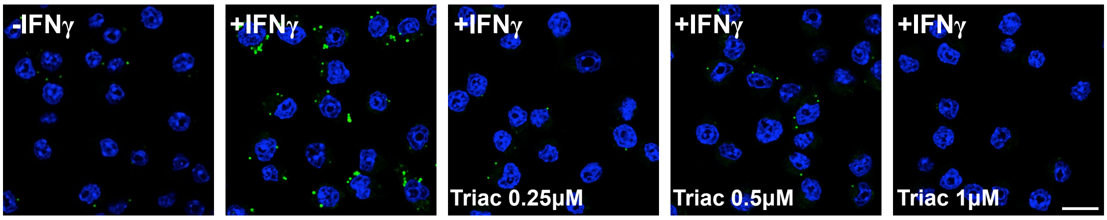

Supplementary Figure 5. Triacsin inhibits lipid droplet accumulation induced by IFNγ. Confocal fluorescence images of Maf-DKO macrophages stained with LipidTOX and DAPI. Cells were activated with IFNγ for 24 hours at the indicated concentrations of triacsin. Data is representative of 2 experiments**.** Scale bar 12.5 μm.

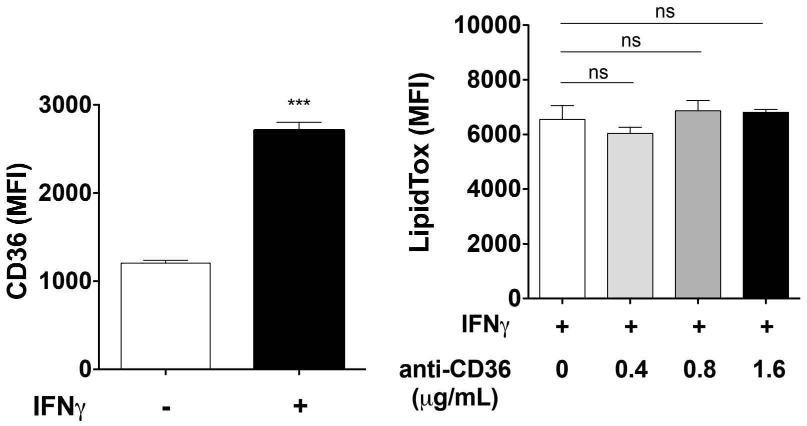

Supplementary Figure 6. Neutral lipid accumulation induced by IFNγ is not dependent on CD36. Surface expression of CD36 (left) in Maf-DKO activated with IFNγ. LipidTOX levels (right) in macrophages activated with IFNγ in the presence of different concentrations of an anti-CD36 blocking antibody (clone 63). Data is representative of 2 experiments.

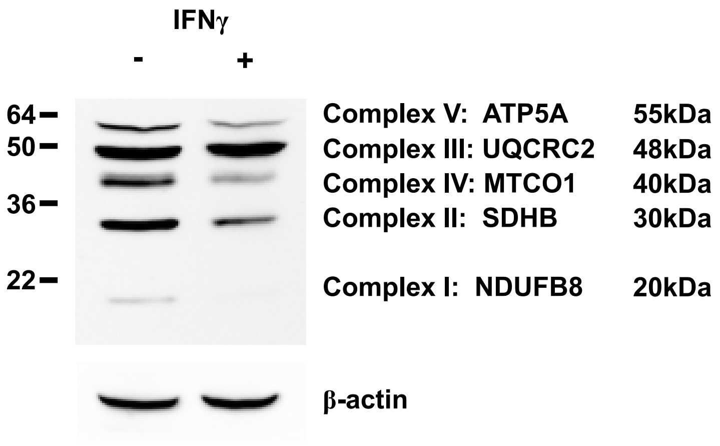

Supplementary Figure 7. IFNγ down-regulates proteins from mitochondrial respiratory complexes. Western blot of mitochondrial respiratory complexes of total protein extracted from Maf-DKO macrophages after incubation for 24 hours in the presence or absence of IFNγ. Results are from one experiment. ATP5A, ATP synthase subunit alpha; UQCRC2, cytochrome b-c1 complex subunit 2; MTCO1, cytochrome oxidase subunit I; SDHB, succinate dehydrogenase complex subunit B; NDUFB8, NADH dehydrogenase 1 beta sub-complex 8.

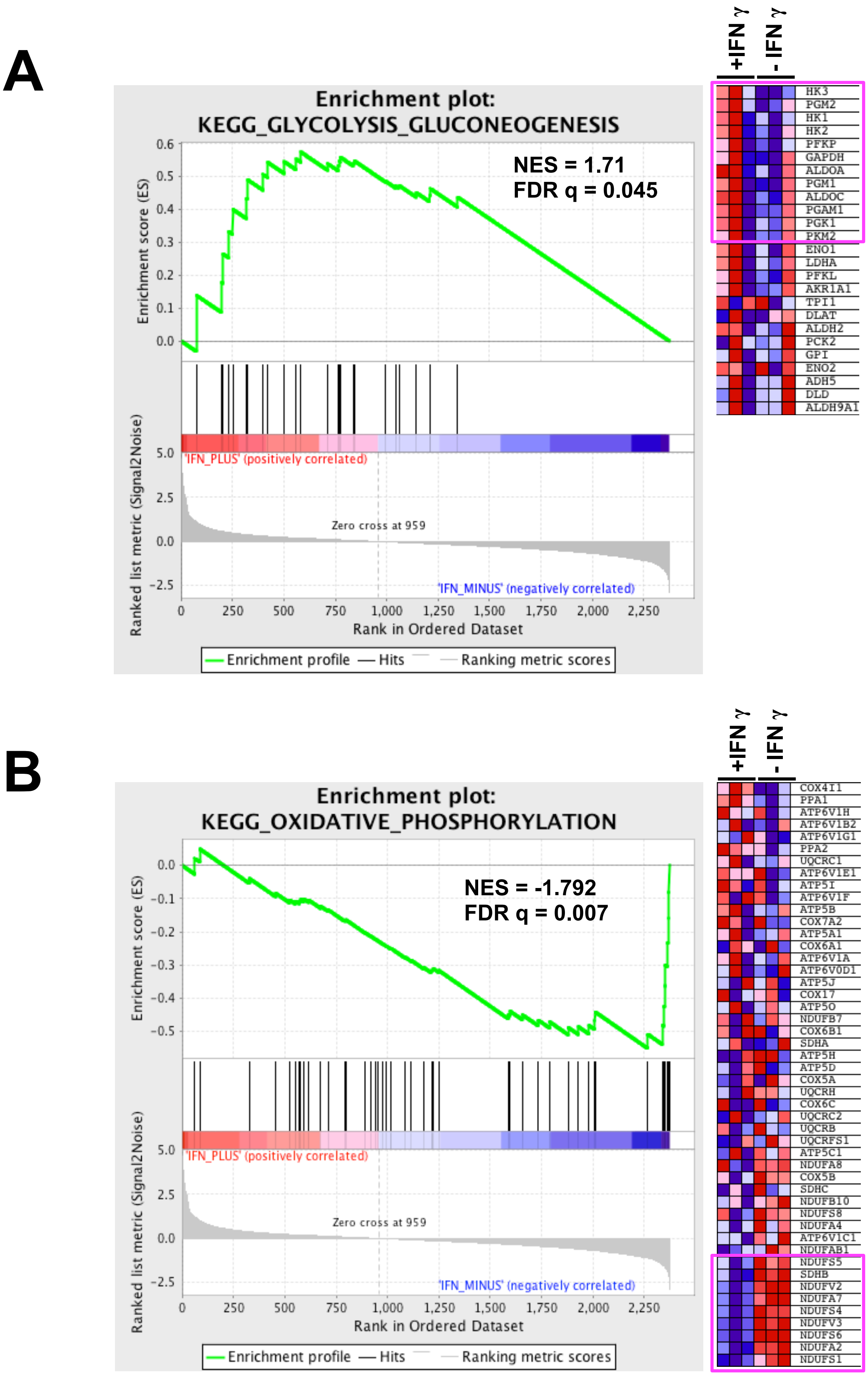
A
+IFN γ
- IFN γ
Enrichment plot:
KEGG_GLYCOLYSIS_GLUCONEOGENESIS
NES = 1.71
FDR q = 0.045
Enrichment score (ES)
'IFN_PLUS' (positively correlated)
Zero cross at 959
'IFN_MINUS' (negatively correlated)
Ranked list metric (Signal2Noise)
Rank in Ordered Dataset
Enrichment profile — Hits — Ranking metric scores
HK3
PGM2
HK1
HK2
PFKP
GAPDH
ALDOA
PGM1
ALDOC
PGAM1
PGK1
PKM2
ENO1
LDHA
PFKL
AKR1A1
TPI1
DLAT
ALDH2
PCK2
GPI
ENO2
ADH5
DLD
ALDH9A1
B
+IFN γ
- IFN γ
Enrichment plot:
KEGG_OXIDATIVE_PHOSPHORYLATION
NES = -1.792
FDR q = 0.007
Enrichment score (ES)
'IFN_PLUS' (positively correlated)
Zero cross at 959
'IFN_MINUS' (negatively correlated)
Ranked list metric (Signal2Noise)
Rank in Ordered Dataset
Enrichment profile — Hits — Ranking metric scores
COX4I1
PPA1
ATP6V1H
ATP6V1B2
ATP6V1G1
PPA2
UQCRC1
ATP6V1E1
ATP5I
ATP6V1F
ATP5B
COX7A2
ATP5A1
COX6A1
ATP6V1A
ATP6V0D1
ATP5J
COX17
ATP5O
NDUFB7
COX6B1
SDHA
ATP5H
ATP5D
COX5A
UQCRH
COX6C
UQCRC2
UQCRB
UQCRFS1
ATP5C1
NDUFA8
COX5B
SDHC
NDUFB10
NDUFS8
NDUFA4
ATP6V1C1
NDUFAB1
NDUFS5
SDHB
NDUFV2
NDUFA7
NDUFS4
NDUFV3
NDUFS6
NDUFA2
NDUFS1

Supplementary Figure 8. Gene set enrichment analysis of representative pathways up- or down-regulated by IFNγ. (A) Running sum plot for the "Glycolysis/gluconeogenesis" and **(B)** "Oxidative phosphorylation" pathways through the ranked list of proteins obtained from the proteome data set. Proteins within each pathway and their relative expression levels are shown. The pink frame identifies proteins contributing to core enrichment. NES, normalized enrichment score; FDR, false discovery rate. Data are normalized by row and originate from 3 independent experiments.

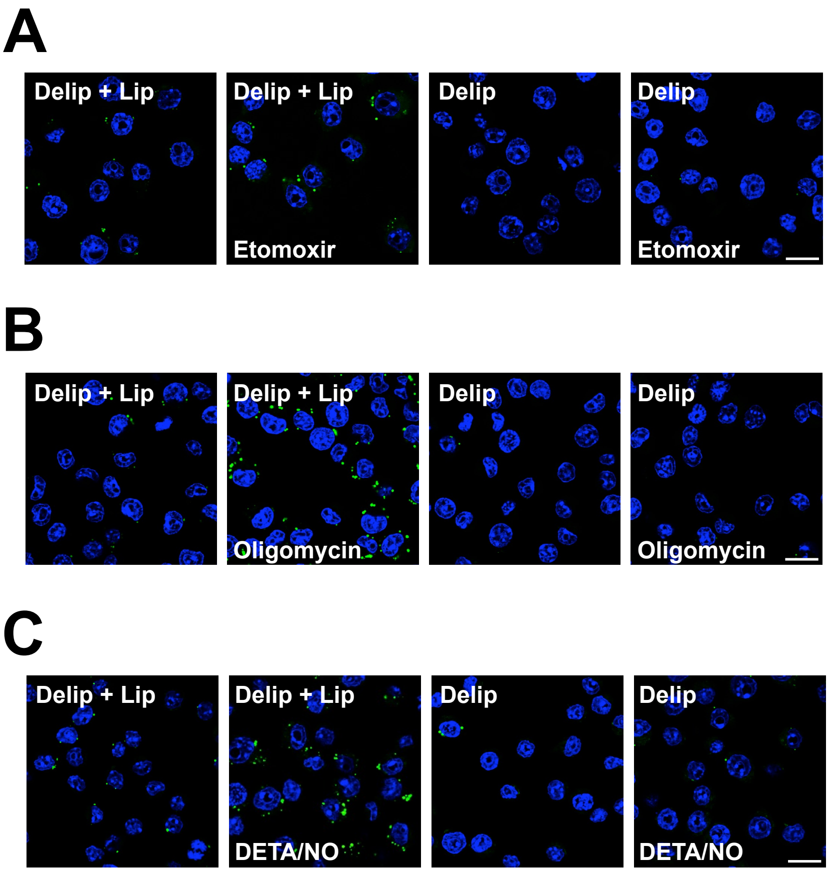

Supplementary Figure 9. Lipid droplet accumulation induced by etomoxir, oligomycin and DETA/NO is dependent on exogenous lipids. Confocal fluorescence images of Maf-DKO macrophages stained with LipidTOX and DAPI. Non-activated cells were incubated for 24 hours in the presence or absence of **(A)** etomoxir, **(B)** oligomycin, and **(C)** DETA/NO; and in medium containing delipidated (Delip) FCS, or delipidated FCS plus lipid mixture (Delip + Lip). Data is representative of 2 experiments**.** Scale bar 12.5 µm.

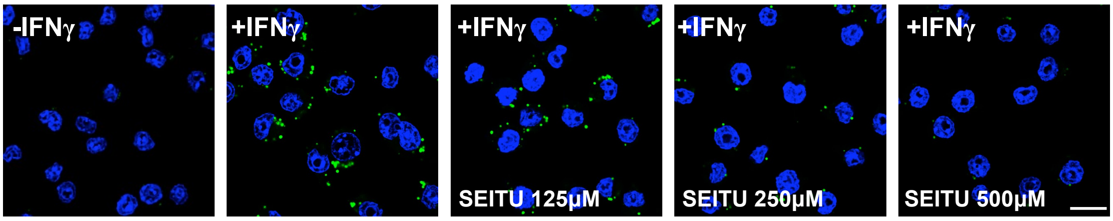

Supplementary Figure 10. SEITU inhibits lipid droplet accumulation induced by IFNγ. Confocal fluorescence images of Maf-DKO macrophages stained with LipidTOX and DAPI. Cells were activated with IFNγ for 24 hours at the indicated concentrations of the iNOS inhibitor SEITU. Data is representative of 2 experiments**.** Scale bar 12.5 µm.
